# Comprehensive Physiotherapeutic Management of Atlas Occipitalization: A Case Report

**DOI:** 10.7759/cureus.55660

**Published:** 2024-03-06

**Authors:** Anandi R Dave, Mitushi A Deshmukh, Siddhant S Deshmukh

**Affiliations:** 1 Department of Physiotherapy, Datta Meghe Institute of Higher Education and Research (Deemed to be University), Wardha, IND; 2 Department of Musculoskeletal Physiotherapy, Ravi Nair Physiotherapy College, Datta Meghe Institute of Higher Education and Research (Deemed to be University), Wardha, IND

**Keywords:** cervical range of motion (rom), muscle strengthening exercises, cervical spine manipulation, conservative therapy, physical therapy rehabilitation, cervical vertebral fusion syndrome, musculoskeletal physiotherapeutic management, cervical pathology, atlas occipitalization

## Abstract

The atlas (C1) and occipital bone at the base of the skull fuse together in atlas occipitalization, an uncommon congenital abnormality. Because it can result in cervical spine instability, nerve impingement, and related symptoms including stiffness, pain, and neurological impairments, it poses a challenging therapeutic problem. We describe the case of a female patient, 27 years old, who had gradually deteriorating neck discomfort, stiffness, and limited cervical mobility for six years prior to presentation. Her symptoms worsened over time despite conservative treatment, so more testing was necessary. Atlas occipitalization, congenital fusion at the C7 and D1 vertebrae, and other related cervical spine pathologies were identified by imaging examinations. The intricacies of atlas occipitalization and related cervical spine pathologies are highlighted in this case study, along with the diagnostic difficulties and interdisciplinary therapeutic strategy needed to address them. To improve cervical range of motion (ROM), lessen discomfort, and improve functional results, the patient underwent a thorough musculoskeletal examination and was given a customized physiotherapeutic intervention.

## Introduction

Atlas occipitalization is an uncommon congenital condition that results in structural changes to the upper cervical spine. It is caused by the fusion of the atlas (C1) with the occipital bone at the base of the skull [1]. The condition provides substantial concerns since it can induce cervical spine instability and related neurological symptoms, even though the incidence in the general population continues to be low [2]. While the precise cause of atlas occipitalization remains unidentified, it is thought to result from atypical embryological development that occurs throughout fetal development [3]. The symptoms of atlas occipitalization can vary widely; some people have no symptoms at all, while others have neck discomfort, stiffness, restricted range of motion (ROM), and neurological abnormalities including tingling or weakness in the upper limbs [4]. The majority of cases are identified in the early stages of adulthood, suggesting that age could have an impact on the symptoms. Moreover, a hereditary component to this illness could exist. However, the precise genetic components are still unclear [5]. Atlas occipitalization could make a big difference in everyday tasks, which may hinder functional mobility and quality of life [6]. Imaging tests of the cervical spine, such as X-rays, computed tomography (CT) scans, or magnetic resonance imaging (MRI), are commonly used in diagnostic evaluations to analyze related cervical spine pathologies and verify the fusion of the atlas with the occipital bone [7].

Physiotherapy is a vital component of atlas occipitalization care, with the goals of reducing symptoms, increasing cervical spine mobility, and improving functional outcomes [8]. Physiotherapists utilize a thorough rehabilitation program that includes manual treatment methods, therapeutic exercises, and patient education to address muscle imbalances, improve posture, and maximize the health of the musculoskeletal system overall. Effective management of pain and muscular spasms can also be achieved with the use of electrotherapeutic methods including transcutaneous electrical nerve stimulation (TENS) and interferential therapy (IFT). In this regard, the case study emphasizes the significance of a multidisciplinary approach to maximize patient outcomes and quality of life by highlighting the clinical presentation, diagnostic problems, and the role of physiotherapy in the management of atlas occipitalization.

## Case presentation

Six years of gradually deteriorating neck discomfort, stiffness, and limited cervical mobility were reported by a 27-year-old female patient. Despite initially seeking relief from a local physician, her symptoms persisted and intensified over time. Two years later, due to escalating pain and intolerable symptoms, she underwent physiotherapy treatment, which provided temporary relief but failed to address the underlying issue. As her symptoms continued to progress, she began experiencing bilateral arm tingling. Seeking further evaluation and management, she presented to the hospital on December 3, 2023. Imaging studies, including X-rays, revealed increased convexity of the left side of the spine, congenital fusion at the C7 and D1 vertebrae, and atlas occipitalization. Subsequent MRI findings confirmed the diagnosis of atlas occipitalization and also revealed increased atlanto-dental interspace. In addition, there are minor disc bulges from C3-C4 to C6-C7 levels that cause the thecal sac to be indented, as well as moderate canal and foraminal constriction that is exacerbated by peridiscal osteophytes. This comprehensive assessment highlighted the complexity of her condition and the need for a multidisciplinary approach to address her symptoms and manage her cervical spine pathology effectively. The patient went for conservative management and, hence, was referred to the musculoskeletal physiotherapy department for further treatment.

Musculoskeletal assessment

In a supine posture, the patient received a thorough musculoskeletal evaluation, exhibiting cooperation and orientation to time, location, and people. The stability of hemodynamics was verified. Using the numeric pain rating scale (NPRS), the patient's pain history exhibited substantial pain levels throughout the upper back and during cervical joint motions, scored 8/10 during activity and 6/10 during rest. Furthermore, there were reports of discomfort and tingling in both upper limbs, which affected daily living activities and cervical range of motion (ROM). As shown in Table 1, the initial active cervical ROM measurement graded ROM as decreased. Palpation revealed discomfort in the soft tissues and spasms in the trapezius muscle. When myotomes were tested to measure peripheral weakness brought on by impingement of the cervical nerve, the results were negative. Table 2 shows that diminished cervical strength was demonstrated by Manual Muscle Testing (MMT). Impingement was verified by the cervical distraction test and the cervical compression test (Spurling's test).

**Table 1:** Range of motion of the cervical spine pre- and post-management R: right, L: left, ROM: range of motion

Joint ROM	Pre-treatment	Post-treatment
Cervical flexion	40°	65°
Cervical extension	0°	10°
Cervical lateral flexion (R)	30°	40°
Cervical lateral flexion (L)	40°	55°

**Table 2:** Manual Muscle Testing of the cervical spine R: right, L: left

Manual Muscle Testing	Pre-treatment	Post-treatment
Cervical flexors	3-	3
Cervical extensors	1	2
Cervical lateral flexors (R)	3-	4
Cervical lateral flexors (L)	3-	4

Investigations

**Figure 1:**
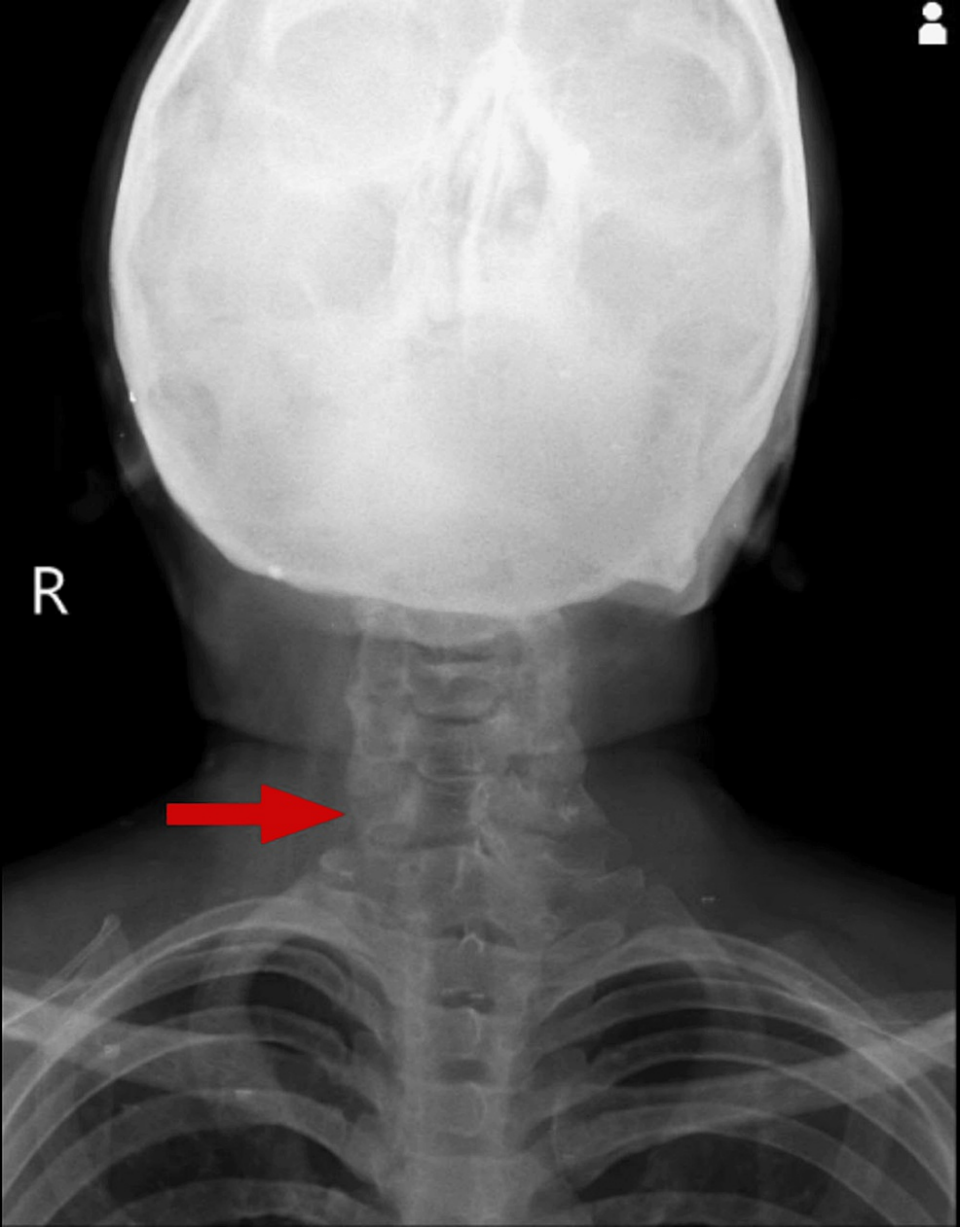
Posterior X-ray of the cervical spine with reduced space between C1 and C7 vertebrae

**Figure 2:**
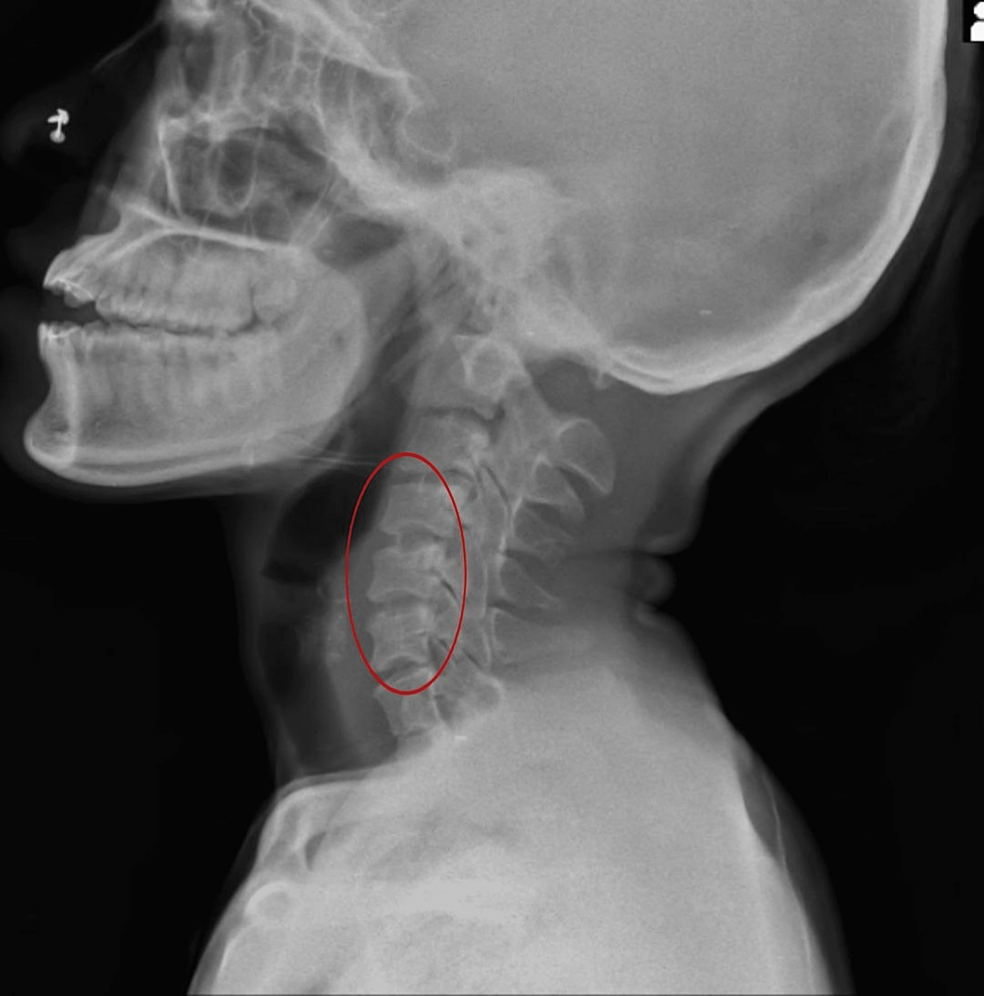
Lateral view of the cervical spine with wedging of cervical vertebrae (osteophyte formation)

Radiological findings revealed reduced intervertebral space between C1 and C7 in posterior view as shown in Figure 1. Wedging of vertebrae and osteophyte formation were observed in lateral view, as shown in Figure 2. Atlas occipitalization was later confirmed by MRI.

Physiotherapeutic intervention

A four-week rehabilitation protocol was planned according to the patient's problem list and goals. A comprehensive approach is mentioned in Table 3.

**Table 3:** Physiotherapy intervention ROM: range of motion, MET: muscle energy technique, IFT: interferential therapy, MFR: myofascial release

Problem list	Goal	Intervention	Duration and dosage
Lack of information about the condition	Patient education	Provide information on posture correction	4 weeks, integrated into each session
		Ergonomic principles and home exercise program	
		Demonstrate proper techniques of exercises	
Neck pain and stiffness	Reduce pain levels	Therapeutic exercises targeting neck muscles	4 weeks, 3 sessions per week 15-20 minutes per session
	Improve cervical ROM	Active and passive stretching of neck muscles	10-15 minutes per session
	Decrease muscle spasms	Manual therapy technique: soft tissue mobilization, MET	
		IFT to relieve pain and muscle spasm	10-12 minutes
Tingling and pain in bilateral arms	Reduce tingling and pain	Neuromuscular re-education exercises	4 weeks, 3 sessions per week
	Improve upper limb function	Strengthening exercises for upper limb muscles	
	Relieve neuromuscular symptoms	Nerve gliding exercises	
		Transcutaneous electrical nerve stimulation	
Reduced cervical ROM	Increase cervical ROM	Cervical mobilization techniques	4 weeks, 3 sessions per week
	Reduce tightness of cervical muscles	Dynamic stretching exercises for neck muscles	
		MFR	
Reduced cervical strength	Improve cervical muscle strength	Isometric exercises targeting cervical muscles	4 weeks, 3 sessions per week
		Progressive resistance exercises for cervical flexors, extensors, lateral flexors, and rotators	

Outcome measures

Outcome measures were incorporated into the assessment protocol, conducted both prior to and following the intervention, as shown in Table 4.

**Table 4:** Outcome measures pre- and post-intervention NDI: Neck Disability Index, NPRS: numerical pain rating scale, RIMT: resistive isometric muscle testing

Outcome measures	Pre-intervention	Post-intervention
NPRS	On rest: 6/10	On rest: 4/10
	On activity: 8/10	On activity: 7/10
NDI	28	15
RIMT	Incomplete, painful	Incomplete, painless

## Discussion

Atlas occipitalization is an uncommon congenital defect characterized by the fusion of the occipital bone at the base of the skull with the atlas (C1), often referred to as assimilation of the atlas or occipitalization of the atlas [9]. A bony bridge between the atlas and the occiput forms as a result of anomalous embryological development during fetal growth, causing this disease [10]. While the exact prevalence of atlas occipitalization in the general population remains unclear, it is considered a relatively uncommon finding, accounting for a small percentage of cervical spine abnormalities [11]. The clinical presentation of atlas occipitalization can vary widely among individuals [4,12]. While some patients may remain asymptomatic, others may experience a spectrum of symptoms related to cervical spine instability and neurological compromise [13]. Common signs and symptoms include neck pain, stiffness, restricted cervical range of motion, and neurological deficits such as tingling, numbness, or weakness in the upper extremities [14]. These symptoms may worsen with physical activity or prolonged periods of neck flexion or extension [15]. Age appears to be a significant factor in the manifestation of symptoms associated with atlas occipitalization, with most cases being diagnosed in early adulthood [16]. Additionally, there may be a genetic predisposition to this condition, although specific genetic factors contributing to its development have yet to be fully elucidated.

Imaging examinations of the cervical spine, such as X-rays, CT scans, or MRIs, are crucial for diagnosing the fusion of the atlas with the occipital bone and evaluating related cervical spine pathologies [17]. These imaging modalities help in visualizing the extent of fusion, evaluating cervical spine alignment, and identifying any additional anomalies or structural abnormalities.

Management of atlas occipitalization typically involves a multidisciplinary approach, with treatment strategies tailored to address individual patient needs and symptoms. While surgical intervention may be considered in cases of severe cervical spine instability or neurological compromise, conservative management approaches, including physiotherapy, are often employed initially to alleviate symptoms and improve functional outcomes. Physiotherapy plays a crucial role in the management of atlas occipitalization, focusing on pain relief, improving cervical spine mobility, and enhancing overall musculoskeletal function [8]. To address muscle imbalances, reduce pain, and improve cervical spine function, therapeutic exercises that target the neck muscles, manual therapy techniques such as soft tissue mobilization, and electrotherapeutic modalities such as transcutaneous electrical nerve stimulation (TENS) or interferential therapy (IFT) are frequently used [18-20].

## Conclusions

The case underscores the importance of a multidisciplinary approach, including physiotherapy, in managing atlas occipitalization. Despite structural abnormalities and cervical spine pathology, coordinated efforts led to effective diagnosis and tailored management. Rehabilitation programs, including exercises and manual therapy, yielded significant improvements in pain levels and cervical range of motion. Patient education empowered active participation in treatment, enhancing long-term self-management. Integrating precision diagnostics and targeted interventions can optimize outcomes for individuals with atlas occipitalization.
